# Correction: Takeuchi et al. Predictive Factors for Poor Outcome following Chemonucleolysis with Condoliase in Lumbar Disc Herniation. *Medicina* 2022, *58*, 1868

**DOI:** 10.3390/medicina59020268

**Published:** 2023-01-30

**Authors:** Shu Takeuchi, Junya Hanakita, Toshiyuki Takahashi, Tomoo Inoue, Manabu Minami, Izumi Suda, Sho Nakamura, Ryo Kanematsu

**Affiliations:** 1 Department of Spinal Disorders Center, Fujieda Heisei Memorial Hospital, Shizuoka 426-8662, Japan; 2 Department of Neurosurgery, Shizuoka General Hospital, Shizuoka 420-8527, Japan; 3 Department of Neurosurgery, Saitama Red Cross Hospital, Saitama 330-0081, Japan

In the original publication [1], there was a mistake in the legend for “**Figure 3.** Change Illustrative example of a patient in the poor group with a calcified disc herniation. T2-weighted sagittal (**A**) and axial (**B**) magnetic resonance images show an L5/S1 disc herniation. Sagittal (**C**) and axial (**D**) computed tomography myelography helped to confirm the calcified disc herniation”. The word change was added by mistake. The correct legend is shown below. The authors state that the scientific conclusions are unaffected. This correction was approved by the Academic Editor. The original publication has also been updated.

“**Figure 3.** Illustrative example of a patient in the poor group with a calcified disc herniation. T2-weighted sagittal (**A**) and axial (**B**) magnetic resonance images show an L5/S1 disc herniation. Sagittal (**C**) and axial (**D**) computed tomography myelography helped to confirm the calcified disc herniation.”
